# Assessment of cognitive impairment after acute cerebral infarction with T1 relaxation time measured by MP2RAGE sequence and cerebral hemodynamic by transcranial Doppler

**DOI:** 10.3389/fneur.2022.1056423

**Published:** 2022-12-06

**Authors:** Hongting Yan, Honghai Chen, Yanzhi Liu, Qiannan Zhang, Yunchu Guo, Yu Fu, Hongling Ren, Hairong Wang, Chun Wang, Yusong Ge

**Affiliations:** ^1^The Department of Neurology, The Second Hospital of Dalian Medical University, Dalian, Liaoning, China; ^2^The Department of Radiology, The Second Hospital of Dalian Medical University, Dalian, Liaoning, China

**Keywords:** cognitive impairment, acute cerebral infarction, MP2RAGE sequence, neuroimaging, microstructural change

## Abstract

**Objective:**

This study aimed to investigate early brain microstructural changes discovered using magnetization-prepared two rapid acquisition gradient echo (MP2RAGE) sequence and cerebral hemodynamic using TCD for cognitive impairment after acute cerebral infarction.

**Methods:**

We enrolled 43 patients with acute cerebral infarction and 21 healthy people in the study, who were subjected to cognitive assessments, the MP2RAGE sequence, and a cerebral hemodynamic examination. A total of 26 brain regions of interest were investigated. Furthermore, we used cerebral hemodynamics to explain brain microstructural changes, which helped us better understand the pathophysiology of cognitive impairment after acute cerebral infarction and guide treatment.

**Results:**

T1 relaxation times in the left frontal lobe, right frontal lobe, right temporal lobe, left precuneus, left thalamus, right hippocampus, right head of caudate nucleus, and splenium of corpus callosum were substantially different across the three groups, which were significantly correlated with neuropsychological test scores. CI group patients had significantly lower cerebral blood flow velocity than those in the N-CI and Normal groups. The receiver operating curve analysis revealed that most T1 relaxation times had high sensitivity and specificity, especially on the right temporal lobe and right frontal lobe. There was a potential correlation between T1 relaxation times and MMSE scores through TCD parameters.

**Conclusion:**

The MP2RAGE sequence can detect alterations in whole brain microstructure in patients with cognitive impairment after acute cerebral infarction. Brain microstructural changes could influence cognitive function through cerebral hemodynamics. T1 relaxation times on the right temporal lobe and the right frontal lobe are expected to be a prospective biomarker of cognitive impairment after acute cerebral infarction.

## Introduction

According to epidemiological studies, 12.2 million new stroke incidents occurred worldwide in 2019 (1). Approximately two-thirds of stroke patients have some degree of cognitive impairment (2). Cognitive impairment following a stroke impairs the patient's capacity to execute daily living activities and increases mortality (3). According to previous research, screening patients for cognitive function during the acute phase of stroke is critical. Early diagnosis and timely care can dramatically improve a patient's prognosis and control disease progression (4). The American Heart Association recommends in its *Guidelines for Adult Stroke Rehabilitation and Recovery* that all patients with acute cerebral infarction be assessed for cognitive impairment before being discharged from the hospital (5).

However, today's cognitive impairment diagnosis is primarily based on the patient's clinical presentation and neuropsychological measures. The scales are susceptible to a number of factors, including subject compliance and the test taker's subjective opinion. Patients with impaired hearing, manual dexterity, or who are bedridden are unable to cooperate with the scale. As a result, the neuropsychological scales have some limitations. Clinicians require a more objective biomarker to diagnose cognitive impairment following acute cerebral infarction.

Many studies in recent years have found a clear correlation between cognitive impairment and neuroimaging (white matter hyperintensities, cortical thickness, and so on) (6–8). However, traditional neuroimaging techniques cannot be quantified. Breakthroughs in neuroimaging may improve the ability to detect cognitive impairment and open up new avenues for early diagnosis. The magnetization-prepared two rapid acquisition gradient echo (MP2RAGE) sequence is a novel quantitative MRI approach. It successfully corrects inhomogeneity in the B1 radio-frequency transmit field and lowers proton contamination as well as T2^*^ contrast (9). It produces quantitative T1 images in order to calculate T1 relaxation time. It is utilized to analyse microstructural alterations in the brain as well as detect diffuse white and gray matter damage in the cranial brain (10). A prior study used relaxation time to detect permanent cerebral ischemia and found that it can predict stroke onset time (11).

The incidence and the recurrence rate of ischemic stroke can often be affected by intracranial arterial lesions (12). The intracranial vascular lesion is the most fundamental cause of cerebrovascular disease, so the detection and evaluation of cerebral vessels and cerebral hemodynamics are significant for preventing and treating cerebrovascular disease. Transcranial Doppler (TCD) is commonly used in clinical practice to examine cerebral hemodynamics, and TCD parameters can reflect various pathological conditions, including atherosclerosis and vascular endothelial dysfunction (13). TCD technique has a high research value in the diagnosis of cerebrovascular diseases and can even identify preclinical cerebral blood flow changes (14, 15). Several investigations have found a clear correlation between cerebral hemodynamic alterations and cognitive impairment (16–18). Many researchers have used TCD to investigate cerebral hemodynamic changes in patients with cognitive impairment. Some researchers found that hemodynamic dysfunction measured by TCD might play a pathogenic role in the development of cognitive impairment also in patients with subcortical ischemic vascular disease (19). It has also been found that the severity of cerebral hemodynamic abnormalities observed by TCD may, to some extent, represent the severity of cognitive impairment (20).

This study aimed to look at microstructural changes at the whole-brain level in individuals with cognitive impairment after an acute cerebral infarction utilizing the MP2RAGE sequence, as well as the relationship between microstructural alterations and cerebral hemodynamics. We hypothesized that the altered brain microstructure measured by the MP2RAGE sequence could provide neuroimaging evidence to assist in the diagnosis of patients with cognitive impairment after acute cerebral infarction, and TCD might be able to provide evidence of cerebral hemodynamic for this altered brain microstructure.

## Materials and methods

### Study participants

This study included 43 patients with acute cerebral infarction, including 23 with cognitive impairment (CI group, mean age 68.91 years, 9 males, and 14 females) and 20 without cognitive impairment (N-CI group, mean age 67.95 years, 12 males, and 8 females) in the Second Hospital of Dalian Medical University. Our institution's Ethics Review Board examined and approved this study procedure. The following were the inclusion criteria for patients with acute cerebral infarction: (1) first acute onset within 7 days of cerebral infarction; (2) symptoms or signs lasting > 24 h; (3) imaging revealing a single ischemic lesion on the relevant side with no intercerebral hemorrhage. The following were the exclusion criteria: (1) recurrent stroke; (2) pre-stroke significant cognitive dysfunction, which was assessed using the informant questionnaire on cognitive decline in the elderly (IQCODE > 3.3) by asking the informant and caregiver; (3) aphasia, dysarthria, hearing impairment, and inability to cooperate with examinations; (4) any other structural brain structure damage detected by MRI; (5) Fazekas classification for white matter hyperintensity > grade 1; (6) a history of alcohol or drug addiction; (7) major folic acid and Vitamin B_12_ abnormalities; (8) pre-existing schizophrenia, severe anxiety, depression, or other mental health disorders; (9) patients with severe disease or severe disease aggravated by vital organ malfunction; (10) patients with metallic materials or other implants in the body that preclude the use of MRI; and (11) patients who lack informed capacity and refuse to sign the informed consent form. Simultaneously, 21 healthy individuals of similar ages were recruited (control group, mean age 65.86 years, 9 males, and 12 females). The following were the inclusion criteria for healthy individuals: (1) matched by gender, age, and education to the CI and N-CI groups; (2) no history of clinical stroke; (3) no neurological dysfunction; (4) cognitive assessment test scores within normal range; (5) MRI studies revealed no brain structural damage; (6) Fazekas classification for white matter hyperintensity ≤ grade 1; (7) no history of alcohol or drug dependency; (8) no substantial abnormalities in folic acid and Vitamin B_12_; (9) no schizophrenia, severe anxiety, depression, or other mental health conditions; (10) no severely advanced disease or severe disease complicated by vital organ dysfunction; (11) no metallic materials or other implants in the body prohibiting the use of MRI; and (12) have the capacity to learn and agree to sign the informed consent form.

### Neuropsychological assessment

A properly trained clinician used neuropsychological measures to assess all patients. The Mini-mental state examination (MMSE), the Montreal cognitive assessment scale (MoCA), and the Activity of daily living scale (ADL) were among the neuropsychological assessment used. MMSE values were used to categorize, with a score of <27 indicating objective cognitive impairment. To determine if respondents had anxiety or depressive disorders, the Hamilton Anxiety (HAMA) and Hamilton Depression (HAMD) scales were employed.

### Magnetic resonance imaging acquisition

All brain MRI scans were performed using a Skyra 3.0 T equipment (Siemens) and a 20-channel head/neck coil. The MP2RAGE sequence took 5 min and 47 s [voxel size=1 mm × 1 mm × 1 mm, the field of view = 256^*^240 mm, repetition time (TR) = 5,000 ms, echo time (TE) = 2.98 ms, TI1 = 700 ms, TI2 = 2,500 ms, flip angle1 = 4°, flip angle2 = 5°, 176 slices]. The MP2RAGE sequence created four sets of images automatically: INV1, INV2, UNI-Images, and T1-Images, and we used the last set of data to do quantitative measurements. No contrast was administered during the MP2RAGE sequence. T1-weighted images, T2-weighted images, T2 fluid-attenuated inversion recovery (FLAIR), and diffusion-weighted imaging (DWI) sequences were also included in the MRI protocol.

### Image processing and analysis

The expert radiologist collected raw MP2RAGE images from patients for post-processing in order to generate quantitative T1 maps. T1 relaxation times were calculated using T1 maps generated by the MP2RAGE sequence. In consideration of the possibility of misidentifying brain regions by using automated whole-brain analysis and our extensive experience in the manual region of interest (ROI) analysis, we selected the latter as a follow-up work. T1 maps were used to manually draw regions of interest (ROI) on the following brain regions: bilateral frontal lobe, bilateral parietal lobe, bilateral temporal lobe, bilateral occipital lobe, bilateral precuneus, bilateral internal capsule, bilateral corona radiata, bilateral centrum semiovale, genu of corpus callosum, splenium of corpus callosum, bilateral hippocampus, bilateral thalamus, bilateral lentiform nucleus, and bilateral head of caudate nucleus. The radiologist chose the central part of the biggest layer, as well as the structures of the upper and lower adjacent layers, and drew ROIs of the same size (10 mm^2^) in the bilateral lobes' symmetrical regions. When drawing ROIs, interfering regions such as the infarct zone, vascular space, cerebral sulcus and gray matter were avoided. The measurements were repeated five times, and the average of the five repeated measurements was used in the statistical analysis.

### Transcranial Doppler ultrasound procedure

A professional sonographer who was blind to the clinical diagnosis performed TCD measurements (TCD 2000S, Chioy, equipped with a 2-MHz probe). The individuals were positioned supine, with the TCD probe situated on the temporal window. The middle cerebral artery (MCA), anterior cerebral artery (ACA), posterior cerebral artery (PCA), basilar artery (BA) and vertebral artery (VA) were probed bilaterally at depths of 45–60 mm, 63–72 mm, and 63–76 mm, respectively, and the systolic velocity (Vs), diastolic velocity (Vd), mean velocity (Vm), pulsatility index (PI) and resistivity index (RI) of each vessel were recorded.

### Statistical analysis

SPSS 11.0 was used for statistical analysis. The data is displayed as mean ± standard deviation (SD). The Shapiro-Wilk test was employed to determine the normality of the data. The association between categorical variables was used to analyse its relationship. Chi-square tests were used to compare categorical differences across groups. To compare the two groups of measures, the independent samples *t*-test was utilized. The quantitative differences between groups were investigated using the one-way analysis of variance (ANOVA). The Bonferront test was used to compare factors among groups that met the homogeneity of variance criteria. *P*-values < 0.05 were deemed statistically significant (with Bonferroni corrections for multiple testing where necessary). Pearson's correlation analysis was used to examine the relationship between two continuous variables. The area under the curve (AUC), specificity, and sensitivity of significant correlation values were assessed individually using receiver operating characteristic (ROC) curves. The discriminatory capacity of measured factors to predict cognitive impairment following an acute cerebral infarction was evaluated using ROC curves. All *P*-values presented are two-tailed. In addition, SPSS 11.0 was adopted to perform the mediation analysis.

## Results

### General characteristics and neuropsychological tests

Table 1 showed that there were no significant differences among the three groups in terms of age, gender, history of hypertension, history of diabetes, history of smoking, history of drinking, duration of education, Body Mass Index (BMI), triglyceride, total cholesterol, low-density lipoprotein, homocysteine or uric acid. Furthermore, there were no significant changes between the CI and N-CI groups in NIHSS (National Institutes of Health Stroke Scale) scores, mRS (Modified Ranking Scale) scores, or the characteristics (side, site, type) and treatments of cerebral infarction (*P* > 0.05).

**Table 1:** General characteristics of the study subjects.

		**CI group** **(*N* = 23)**	**N-CI group** **(*N* = 20)**	**NORM group (*N* = 21)**	***X*** **^2^/*T*/*F***	***p***
Age (years)		68.91 ± 8.43	67.95 ± 4.17	65.86 ± 4.83	1.37	0.26
Gender	Male	9 (39.1)	12 (60.0)	9 (42.9)	2.07	0.36
	Female	14 (60.9)	8 (40.0)	12 (57.1)		
Hypertension	No	3 (13.0)	8 (40.0)	4 (19.0)	4.67	0.10
	Have	20 (87.0)	12 (60.0)	17 (81.0)		
Diabetes mellitus	No	10 (43.5)	7 (35.0)	5 (23.8)	1.89	0.39
	Have	13 (56.5)	13 (65.0)	16 (76.2)		
History of smoking	No	17 (73.9)	10 (50.0)	16 (76.2)	3.92	0.14
	Have	6 (26.1)	10 (50.0)	5 (23.8)		
History of drinking	No	20 (87.0)	16 (80.0)	19 (90.5)	0.96	0.62
	Have	3 (13.0)	4 (20.0)	2 (9.5)		
Duration of education	< 9	6 (26.1)	6 (30.0)	6 (28.6)	0.08	0.96
	≥9	17 (73.9)	14 (70.0)	15 (71.4)		
Fazekas	Grade 0	16 (69.6)	15 (75.0)	15 (71.4)	0.08	0.93
	Grade 1	7 (30.4)	5 (25.0)	6 (28.6)		
Size of the infarction	0–20 mm	17 (73.9)	15 (75.0)	–	0.25	0.88
	20–40 mm	4 (17.4)	4 (20.0)	–		
	>40 mm	2 (8.7)	1 (5.0)	–		
Side of the infarction	Left	11 (47.8)	9 (45.0)	–	0.03	0.86
	Right	12 (52.2)	11 (55.0)	–		
Site of the infarction	Frontal lobe	5 (21.7)	5 (21.7)	–	9.17	0.33
	Parietal lobe	5 (25.0)	5 (25.0)	–		
	Temporal lobe	3 (13.0)	1 (5.0)	–		
	Occipital lobe	2 (8.7)	0	–		
	Thalamus	2 (8.7)	3 (15.0)	–		
	Pons	2 (8.7)	0	–		
	Medulla oblongata	1 (4.3)	2 (10.0)	–		
	Cerebellum	2 (8.7)	0	–		
	Corona radiata	1 (4.3)	4 (20.0)	–		
Duration	(Apopiecticus insultus to examination)	45.39 ± 11.78	45.65 ± 11.92	–	0.01	0.94
TOAST classification	Large-artery atherosclerotic	17 (73.9)	16 (80.0)	–	0.21	0.65
	Cardioembolism	0	0	–		
	Small-vessel disease	6 (26.1)	4 (20.0)	–		
	Other and undetermined etiologies	0	0	–		
Intravenous thrombolysis	No	19 (82.6)	16 (80.0)	–	0.05	0.83
	Have	4 (17.4)	4 (20.0)	–		
Antiplatelet aggregation		23 (100.00)	20 (100.00)	–		–
Intensive lipid lowering		23 (100.00)	20 (100.00)	–		–
NIHSS		1.69 ± 1.18	1.70 ± 1.12	–	−0.01	0.99
mRS		1.13 ± 0.69	1.05 ± 0.51	–	0.43	0.67
BMI		24.1 ± 3.05	24.85 ± 2.57	26.14 ± 3.14	2.29	0.08
TG (mmol/L)		1.47 ± 0.34	1.68 ± 0.38	1.44 ± 0.32	2.77	0.07
TC (mmol/L)		4.9 ± 1.17	4.26 ± 1.54	4.52 ± 0.83	1.54	0.22
LDL-C (mmol/L)		3.02 ± 0.84	3.02 ± 1.06	2.7 ± 0.66	0.96	0.39
HCY (umol/L)		12.79 ± 3.95	11.64 ± 3.87	11.21 ± 3.16	1.09	0.34
UA (umol/L)		300.53 ± 126.29	321.64 ± 78.84	318.47 ± 107.28	0.25	0.78

CI, cognitive impairment; N-CI, no-cognitive impairment; NORM, normal; TOAST, Trial of Org 10172 in Acute Stroke Treatment; NIHSS, National Institutes of Health Stroke Scale; mRS, Modified Ranking Scale; BMI, Body Mass Index; TG, Triglyceride; TC, Total cholesterol; LDL-C, Low-density lipoprotein; HCY, Homocysteine; UA, Uric Acid; Statistical significance was set to *p* < 0.05.

There were significant differences in MMSE, MoCA, and ADL scores among the three groups (*p* < 0.01). The MMSE and MoCA scores were lowest in the CI group and highest in the Normal group. The ADL scores of the CI group were greater than those of the Normal and N-CI groups. The HAMA and HAMD scores did not differ significantly across the three groups (Table 2).

**Table 2:** Neuropsychological tests of the study subjects.

	**CI group (*N* = 23)**	**N-CI group (*N* = 20)**	**NORM group (*N* = 21)**	***F***	***p***
MMSE	19.61 ± 4.68^*^#	27.9 ± 1.12	28.48 ± 1.25	61.95	< 0.01
MoCA	15.65 ± 5.49^*^#	27.4 ± 0.6	27.48 ± 0.81	91.38	< 0.01
ADL	44.74 ± 16.03^*^#	24.55 ± 4.07	20 ± 0.00	39.29	< 0.01
HAMA	3.69 ± 1.58	4.00 ± 1.62	3.71 ± 2.02	0.20	0.82
HAMD	4.13 ± 1.89	3.40 ± 1.53	3.52 ± 1.80	1.09	0.34

MMSE, Mini-Mental State Examination; MoCA, Montreal cognitive assessment; ADL, Activity of daily living; HAMA, Hamilton Anxiey Scale; HAMD, Hamilton Depression Scale;

* *p* < 0.01 vs. NORM group, #*p* < 0.01 vs. N-CI group.

### Comparisons of T1 relaxation times among the three groups

T1 relaxation times in the left frontal lobe, right frontal lobe, right temporal lobe, left precuneus, left thalamus, right hippocampus, right head of caudate nucleus, and splenium of corpus callosum (*P* < 0.05) were substantially different across the three groups.

The following ROIs had significant differences in T1 relaxation times between the CI and Normal groups (*P* < 0.05): left frontal lobe, right frontal lobe, left parietal lobe, left temporal lobe, right temporal lobe, left praecuneus, right praecuneus, right internal capsule, left hippocampus, right hippocampus, left thalamus, left head of the caudate nucleus, right head of caudate nucleus, right lentiform nucleus, and splenium of the corpus callosum.

T1 relaxation times for each ROI differed significantly between the N-CI and Normal groups (*P* < 0.05): left frontal lobe, right frontal lobe, left temporal lobe, right temporal lobe, left precuneus, right centrum semiovale, left hippocampus, right hippocampus, left thalamus, left head of the caudate nucleus, right head of caudate nucleus, splenium of the corpus callosum.

**Figure 1:**
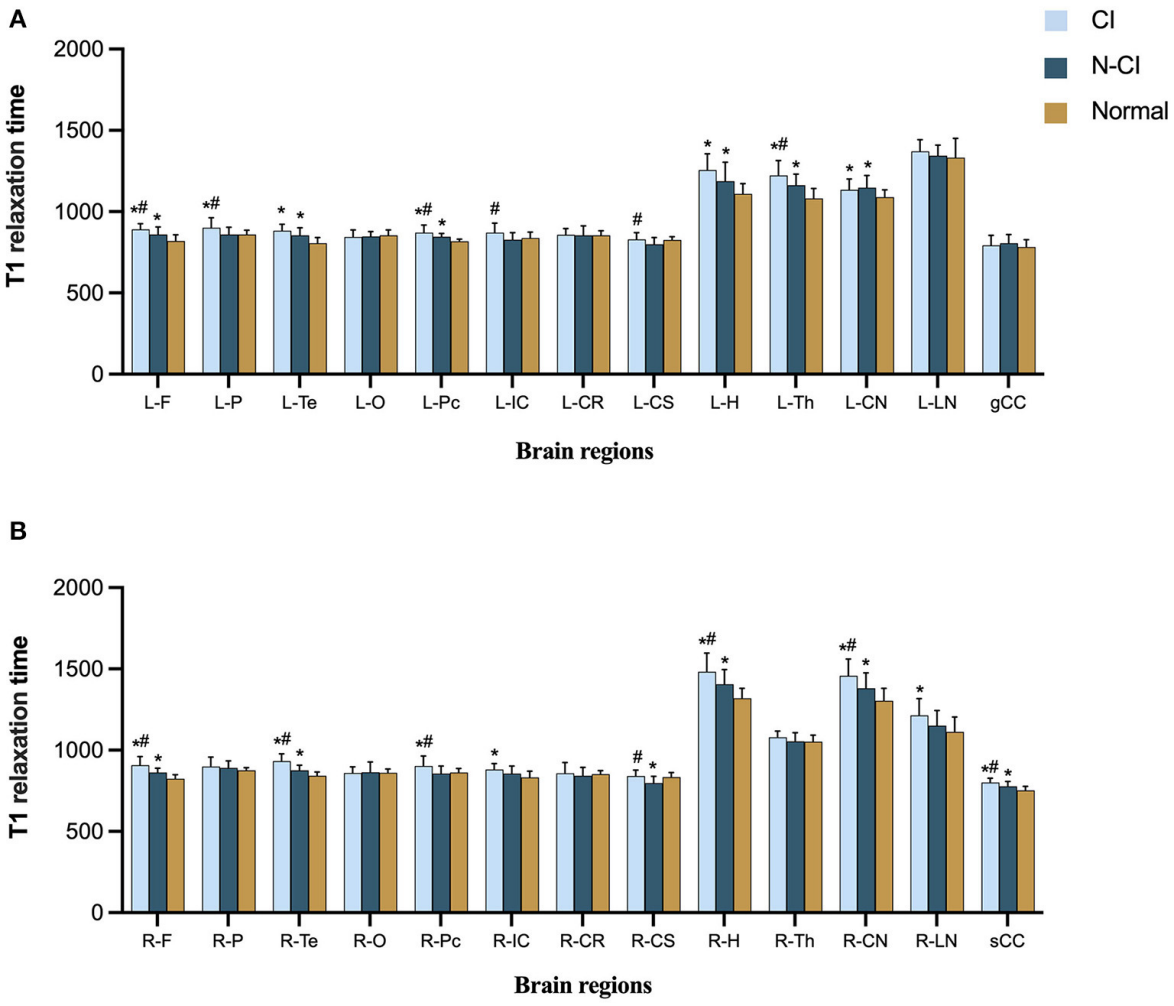
Means (±SD) of T1 relaxation times in each ROI, for each study group. **(A)** Left hemisphere and genu of corpus callosum; **(B)** right hemisphere and splenium of corpus callosum. CI patients had significantly higher T1 relaxation times in most ROIs than the N-CI and Normal groups. ROIs: L-F, left frontal lobe; L-P, left parietal lobe; L-Te, left temporal lobe; L-O, left occipital lobe; L-Pc, left precuneu; L-IC, left internal capsule; L-CR, left corona radiata; L-CS, left centrum semiovale; L-H, left hippocampus; L-Th, left thalamus; L-CN, left corona radiata; L-LN, left lentiform nucleus; R-F, right frontal lobe; R-P, right parietal lobe; R-Te, right temporal lobe; R-O, right occipital lobe; R-Pc, right precuneu; R-IC, right internal capsule; R-CR, right corona radiata; R-CS, right centrum semiovale; R-H, right hippocampus; R-Th, right thalamus; R-CN, right corona radiata; R-LN, right lentiform nucleus; gCC, genu of corpus callosum; sCC, splenium of corpus callosum.*p < 0.05 vs. Normal group, #*p* < 0.05 vs. N-CI group.

**Figure 2:**
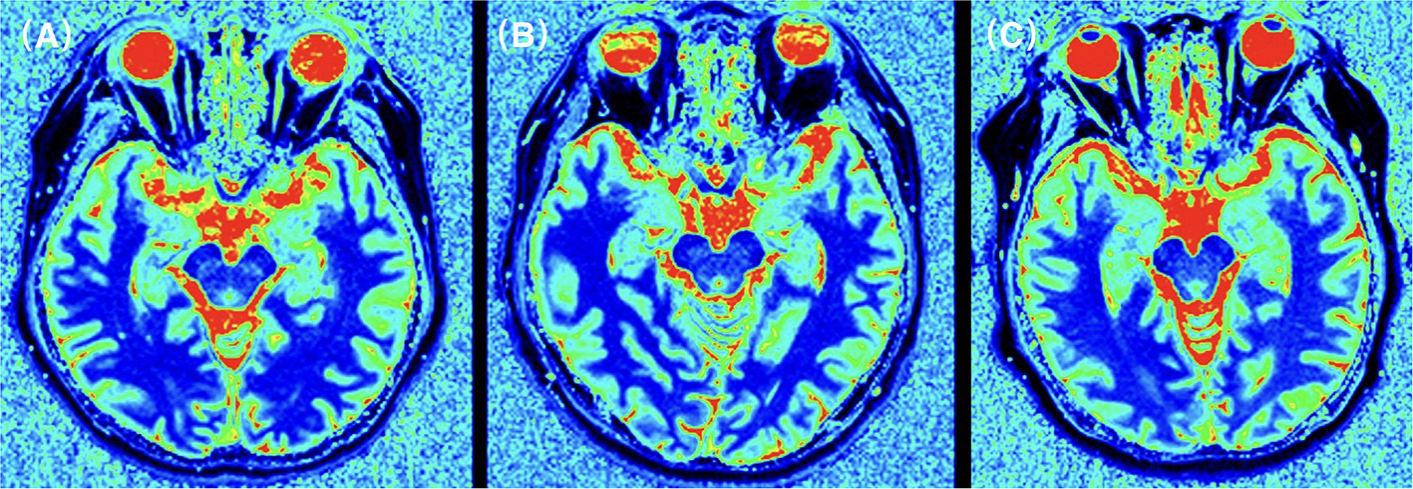
T1 relaxation times for the temporal lobe in an CI patient **(A)**, a N-CI patient **(B)**, and a healthy control **(C)**. T1 relaxation maps was generated for each subject. ROIs were manually plotted on these maps and parameter values were recorded. The results showed that the T1 relaxation times in the frontal lobe of the CI group were higher than those in the N-CI and normal groups (*p* < 0.05).

T1 relaxation times differed significantly between the N-CI and CI groups in the following ROIs (*P* < 0.05): left frontal lobe, right frontal lobe, left parietal lobe, right temporal lobe, left praecuneus, right praecuneus, left internal capsule, left centrum semiovale, right centrum semiovale, right hippocampus, left thalamus, right head of caudate nucleus, splenium of corpus callosum (Figures 1, 2).

### Abnormalities in TCD parameters

Table 3 showed significant differences in Left MCA Vd, Left MCA Vm, Left MCA PI, Left MCA RI, Right MCA Vd, Right MCA Vm Left ACA Vd, Left ACA Vm, Left ACA PI, Right ACA Vs, Right ACA RI, Right PCA Vd, BA Vd, BA Vm, BA PI, and BA RI, among the three groups.

**Table 3:** TCD parameters of the study subjects.

	**CI group (*N* = 23)**	**N-CIgroup (*N* = 20)**	**Normal group (*N* = 21)**	***F***	***p***
Left MCA Vs	109.09 ± 25.38	126.75 ± 48.77	127.33 ± 27.1	1.96	0.15
Left MCA Vd	33.3 ± 8.28^*^	41.85 ± 18.1	49.81 ± 12.67	8.35	**< 0.01**
Left MCA Vm	58.57 ± 11.17^*^	70.15 ± 26.31	75.65 ± 16.28	4.84	**0.01**
Left MCA PI	1.29 ± 0.29^*^	1.21 ± 0.26	1.03 ± 0.19	5.86	**< 0.01**
Left MCA RI	0.69 ± 0.79^*^	0.66 ± 0.08	0.61 ± 0.06	6.04	**< 0.01**
Right MCA Vs	109.52 ± 30.74	118.25 ± 38.36	130.24 ± 35.83	1.94	0.15
Right MCA Vd	42.78 ± 14.34^*^	43.85 ± 16.34	56.67 ± 20.17	4.37	**0.02**
Right MCA Vm	65.03 ± 16.62^*^	68.65 ± 22.41	81.19 ± 22.92	3.62	**0.03**
Right MCA PI	1.04 ± 0.34	1.09 ± 0.23	0.93 ± 0.25	1.91	0.16
Right MCA RI	0.60 ± 0.12	0.63 ± 0.08	0.56 ± 0.11	1.78	0.18
Left ACA Vs	65.53 ± 21.23	69.53 ± 13.02	72.7 ± 16.51	0.93	0.40
Left ACA Vd	21.07 ± 8.19^*^	26.95 ± 5.8	28.83 ± 6.14	7.71	**< 0.01**
Left ACA Vm	35.89 ± 11.56^*^	41.14 ± 6.36	43.45 ± 7.92	4.09	**0.02**
Left ACA PI	1.25 ± 0.31^*^	1.04 ± 0.27	1 ± 0.28	4.60	**0.01**
Left ACA RI	0.60 ± 0.14	0.60 ± 0.11	0.63 ± 0.80	0.79	0.46
Right ACA Vs	64.69 ± 12.89^*^	66.2 ± 11.44	74.93 ± 10.78	4.71	**0.01**
Right ACA Vd	26.28 ± 7.68	25.29 ± 6.94	27.31 ± 6.51	0.45	0.64
Right ACA Vm	38.42 ± 6.52	39.59 ± 7.15	43.18 ± 6.76	2.87	0.06
Right ACA PI	1.04 ± 0.35	1.03 ± 0.31	1.12 ± 0.24	0.55	0.58
Right ACA RI	0.67 ± 0.10^*^	0.60 ± 0.10	0.59 ± 0.10	3.73	**0.03**
Left PCA Vs	53.6 ± 21.66	56.42 ± 25.17	68.84 ± 23.5	2.58	0.08
Left PCA Vd	21.47 ± 11.08	21.54 ± 10.4	27.92 ± 12.41	2.27	0.11
Left PCA Vm	32.18 ± 14	33.16 ± 14.7	41.56 ± 15.35	2.64	0.08
Left PCA PI	1.04 ± 0.28	1.06 ± 0.24	1.02 ± 0.28	0.11	0.90
Left PCA RI	0.61 ± 0.13	0.61 ± 0.08	0.60 ± 0.92	0.19	0.83
Right PCA Vs	60.72 ± 20.74	56.45 ± 15.04	65.05 ± 21.04	1.02	0.37
Right PCA Vd	24.83 ± 10.14	18.66 ± 4.73^*^	26.88 ± 10.74	4.57	**0.01**
Right PCA Vm	36.8 ± 12.71	31.26 ± 6.5	39.6 ± 12.59	2.99	0.06
Right PCA PI	0.99 ± 0.29	1.2 ± 0.33	0.99 ± 0.34	2.89	0.06
Right PCA RI	0.59 ± 0.11	0.65 ± 0.11	0.58 ± 0.13	2.57	0.09
BA Vs	56.66 ± 13.01	63.39 ± 27.14	65.89 ± 20.62	1.17	0.32
BA Vd	15.85 ± 3.62^*^#	24.63 ± 10.34	24.98 ± 10.38	8.06	**< 0.01**
BA Vm	29.45 ± 5.14^*^#	37.55 ± 15.4	38.62 ± 12.07	4.24	**0.02**
BA PI	1.37 ± 0.31^*^#	1.03 ± 0.26	1.08 ± 0.35	7.60	**< 0.01**
BA RI	0.71 ± 0.09^*^#	0.6 ± 0.10	0.61 ± 0.13	6.53	**< 0.01**
Left VA Vs	35.08 ± 9.57	44.27 ± 16.51	41.52 ± 9.86	3.23	0.05
Left VA Vd	14.11 ± 4.44	17.25 ± 9.23	16.35 ± 5.58	1.30	0.28
Left VA Vm	21.1 ± 5.43	26.25 ± 10.6	24.74 ± 6.48	2.58	0.08
Left VA PI	1.00 ± 0.34	1.09 ± 0.49	1.05 ± 0.28	0.28	0.76
Left VA RI	0.59 ± 0.12	0.61 ± 0.14	0.61 ± 0.10	0.20	0.80
Right VA Vs	32.63 ± 15.85	39.23 ± 26.73	41.41 ± 14.36	1.22	0.30
Right VA Vd	12.4 ± 4.48	15.74 ± 8.62	15.55 ± 6.8	1.70	0.19
Right VA Vm	19.14 ± 7.89	23.57 ± 14.14	24.17 ± 8.83	1.51	0.23
Right VA PI	1.02 ± 0.31	0.97 ± 0.31	1.08 ± 0.28	0.62	0.54
Right VA RI	0.59 ± 0.11	0.58 ± 0.13	0.62 ± 0.09	0.77	0.47

Left MCA, left middle cerebral artery; Right MCA, right middle cerebral artery; Left ACA, left anterior cerebral artery; Right ACA, right anterior cerebral artery; Left PCA, left posterior cerebral artery; Right PCA, right posterior cerebral artery; BA, basilar artery; Left VA, left vertebral artery; Right VA, right vertebral artery; Vs, systolic velocity; Vd, diastolic velocity; Vm, mean velocity; PI, pulsatility index; RI, resistivity index.

* *p* < 0.05 vs. Normal group, #*p* < 0.05 vs. N-CI group.

The bold values mean *p* < 0.05.

There were significant variations in TCD parameters between the CI, and the Normal groups were as follows (*P* < 0.05): Left MCA Vd, Left MCA Vm, Left MCA PI, Left MCA RI, Right MCA Vd, Right MCA Vm, Left ACA Vd, Left ACA Vm, Right ACA RI, Left ACA PI, Right ACA Vs, BA Vd, BA Vm, BA PI, and BA RI.

There was a significant variation in Right PCA Vd between the N-CI and Normal groups (*P* < 0.05).

### Correlation analysis between T1 relaxation times and neuropsychological tests

The examination of T1 relaxation times revealed a total of 8 ROIs that differed considerably, as indicated above. T1 relaxation times were thought to be helpful in assessing the pathological alterations in brain tissue associated with cognitive impairment in patients with acute cerebral infarction. As a result, we did a follow-up correlation study for T1 relaxation times and Neuropsychological Tests.

T1 relaxation times in the left frontal lobe, right frontal lobe, right temporal lobe, left praecuneus, right hippocampus, left thalamus, right head of caudate nucleus, and splenium of the corpus callosum were significantly negatively correlated with MMSE scores, significantly negatively correlated with MoCA scores, and significantly positively correlated with ADL scores. The most robust correlation coefficient was found between T1 relaxation times in the right frontal lobe and MMSE scores (full Pearson's coefficient values are included in Table 4).

**Table 4:** Pearson's correlations of T1 relaxation times with neuropsychological tests.

	**L-F**	**R-F**	**R-Te**	**L-Pc**	**sCC**	**R-H**	**L-Th**	**R-CN**
MMSE	−0.40^**^	−0.70^**^	−0.58^**^	−0.43^**^	−0.45^**^	−0.36^**^	−0.58^**^	−0.56^**^
MoCA	−0.44^**^	−0.68^**^	−0.58^**^	−0.42^**^	−0.45^**^	−0.39^**^	−0.55^**^	−0.63^**^
ADL	0.37^**^	0.49^**^	0.64^**^	0.43^**^	0.42^**^	0.33^**^	0.53^**^	0.37^**^

MMSE, Mini-Mental State Examination; MoCA, Montreal cognitive assessment; ADL, Activity of daily living; L-F, left frontal lobe; R-F, right frontal lobe; R-Te, right temporal lobe; L-Pc, left precuneu; sCC, splenium of corpus callosum; R-H, right hippocampus; L-Th, left thalamus; R-CN, right corona radiata;

** *p* < 0.01.

### Correlation analysis between TCD parameters and neuropsychological tests

Neuropsychological scores, except for Left MCA PI, Left MCA RI, Right MCA Vd, Right MCA Vm, Right ACA Vs and BA Vm (Table 5), all cerebral hemodynamic indicators with statistical significance, were substantially linked with cognitive scores.

**Table 5:** Pearson's correlations between TCD parameters and neuropsychological tests.

	**MMSE**	**MoCA**	**ADL**
Left MCA Vd	0.31^*^	0.32^*^	−0.40^**^
Left MCA Vm	0.29^*^	0.27^*^	−0.32^*^
Left MCA PI	−0.16	−0.22	0.34^*^
Left MCA RI	−0.17	−0.22	0.34^**^
Right MCA Vd	0.11	0.11	−0.22
Right MCA Vm	0.19	0.16	−0.21
Left ACA Vd	0.45^**^	0.51^**^	−0.42^**^
Left ACA Vm	0.40^**^	0.44^**^	−0.34^**^
Left ACA PI	−0.27^*^	−0.33^**^	0.33^**^
Right ACA Vs	0.13	0.17	−0.1
Right ACA RI	−0.26^*^	−0.30^*^	0.28
BA Vd	0.34^**^	0.38^**^	−0.42^**^
BA Vm	0.22	0.24	−0.36^**^
BA PI	−0.41^**^	−0.45^**^	0.34^**^
BA RI	−0.38^**^	−0.41^**^	0.33^**^

MMSE, Mini-Mental State Examination; MoCA, Montreal cognitive assessment; L-F, left frontal lobe; R-F, right frontal lobe; R-Te, right temporal lobe; L-Pc, left precuneu; sCC, splenium of corpus callosum; R-H, right hippocampus; L-Th, left thalamus; R-CN, right corona radiata; Left MCA, left middle cerebral artery; Right MCA, right middle cerebral artery; Left ACA, left anterior cerebral artery; Right ACA, right anterior cerebral artery; BA, basilar artery; Vs, systolic velocity; Vd, diastolic velocity; Vm, mean velocity; PI, pulsatility index; RI, resistivity index.

** *p* < 0.01;

* *p* < 0.05.

### Correlation analysis between T1 relaxation times and TCD parameters

To determine if cerebral hemodynamic variations can partially explain brain microstructure changes. For the correlation study, we chose T1 relaxation times and TCD parameters that differed statistically across the three groups. T1 relaxation times in most ROIs, save the left praecuneus, were strongly linked with TCD parameters, as shown in Table 6.

**Table 6:** Pearson's correlations between T1 relaxation times and TCD parameters.

	**L-F**	**R-F**	**R-Te**	**L-Pc**	**R-H**	**L-Th**	**R-CN**	**sCC**
Left MCA Vd	−0.16	−0.24	−0.37^**^	−0.19	−0.29^*^	−0.36^**^	−0.22	−0.13
Left MCA Vm	−0.03	−0.16	−0.38^*^	−0.15	−0.20	−0.31^*^	−0.19	−0.14
Left MCA PI	0.33^**^	0.27^*^	0.29^*^	0.16	0.28^*^	0.25^*^	0.15	0.06
Left MCA RI	0.34^**^	0.27^*^	0.30^*^	0.16	0.27^*^	0.25^*^	0.15	0.07
Right MCA Vd	−0.33	−0.19	−0.34	−0.19	−0.20	−0.14	−0.26	−0.30
Right MCA Vm	−0.23	−0.22	−0.36^**^	−0.21	−0.16	−0.14	−0.24	−0.28
Left ACA Vd	−0.25^*^	−0.46^**^	−0.37^**^	−0.23	−0.18	−0.41^**^	−0.31^*^	−0.25^*^
Left ACA Vm	−0.21	−0.46^**^	−0.24	−0.19	−0.14	−0.38^**^	−0.33^**^	−0.22
Left ACA PI	0.20	0.23	0.34^**^	0.21	0.17	0.21	0.11	0.16
Right ACA Vs	−0.17	−0.21	−0.26^*^	−0.24	−0.19	−0.09	−0.20	−0.21
Right ACA RI	0.19	0.21	0.30^*^	0.17	0.13	0.21	0.07	0.15
BA Vd	−0.27^*^	−0.37^**^	−0.33^**^	−0.16	−0.26^*^	−0.21	−0.18	−0.15
BA Vm	−0.12	−0.26^*^	−0.32^**^	−0.14	−0.28^*^	−0.17	−0.05	−0.14
BA PI	0.42^**^	0.39^**^	0.20	0.10	0.05	0.21	0.31^*^	0.11
BA RI	0.43^**^	0.37^**^	0.21	0.12	0.08	0.18	0.29^*^	0.12

L-F, left frontal lobe; R-F, right frontal lobe; R-Te, right temporal lobe; L-Pc, left precuneu; sCC, splenium of corpus callosum; R-H, right hippocampus; L-Th, left thalamus; R-CN, right corona radiata; Left MCA, left middle cerebral artery; Right MCA, right middle cerebral artery; Left ACA, left anterior cerebral artery; Right ACA, right anterior cerebral artery; BA, basilar artery; Vs, systolic velocity; Vd, diastolic velocity; Vm, mean velocity; PI, pulsatility index; RI, resistivity index.

** *p* < 0.01;

* *p* < 0.05.

### Receiver operating characteristic curve analysis

**Figure 3:**
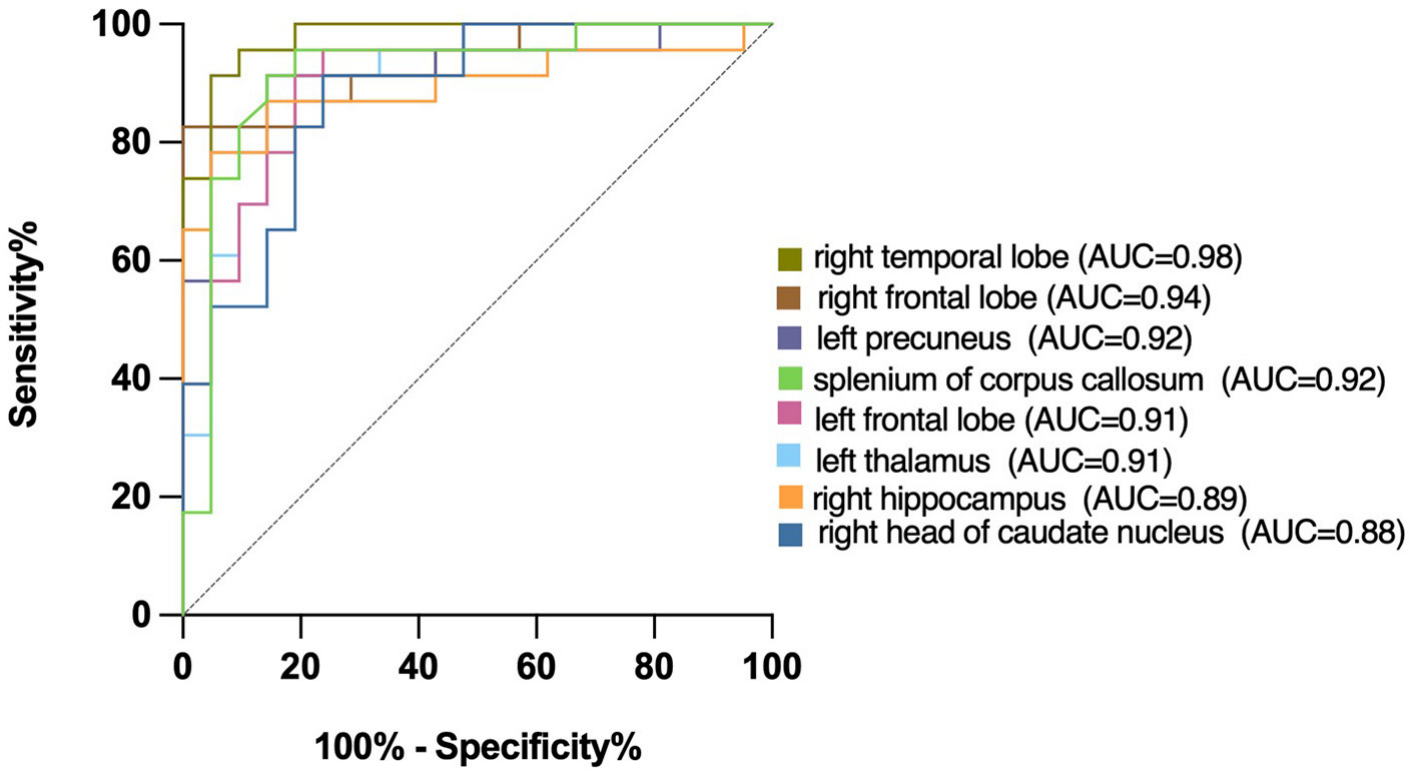
Receiver operating characteristic (ROC) curves for T1 relaxation times, distinguishing CI group from Normal group. T1 relaxation time in the right temporal lobe was identified as a good individual discriminator of cognitive impairment after acute cerebral infarction from healthy controls using ROC analysis; at the optimal cut-off T1 relaxation time of 887.4, sensitivity and specificity were 91.30 and 95.24%, respectively, with an AUC value of 0.98.

We plotted the ROC curve using the T1 relaxation time in all the ROIs that showed a significant effect. Table 7 showed the AUC for the ability of T1 relaxation time to distinguish between healthy controls and patients with cognitive impairment after acute cerebral infarction. T1 relaxation time in the right temporal lobe was identified as an excellent individual discriminator of cognitive impairment after acute cerebral infarction from healthy controls using ROC analysis; T1 relaxation time of 887.4, sensitivity and specificity were 91.30 and 95.24%, respectively, with an AUC value of 0.98 (Figure 3).

**Table 7:** Coordinate points of receiver operating characteristic (ROC) curve.

**(CI vs. normal)**	**AUC**	**Threshold**	**Sensitivity**	**Specificity**
L-F	0.91	853.2	91.30%	85.71%
R-F	0.94	860.3	82.61%	100.00%
R-Te	0.98	887.4	91.30%	95.24%
L-Pc	0.92	827.1	91.30%	85.71%
R-H	0.89	1,406	78.26%	95.24%
L-Th	0.91	1,117	91.30%	80.95%
R-CN	0.88	1,332	91.30%	76.19%
sCC	0.92	771.8	91.30%	85.71%

AUC, area under the curve; L-F, left frontal lobe; R-F, right frontal lobe; R-Te, right temporal lobe; L-Pc, left precuneu; R-H, right hippocampus; L-Th, left thalamus; R-CN, right corona radiata; sCC, splenium of corpus callosum.

### Mediation analysis

**Figure 4:**
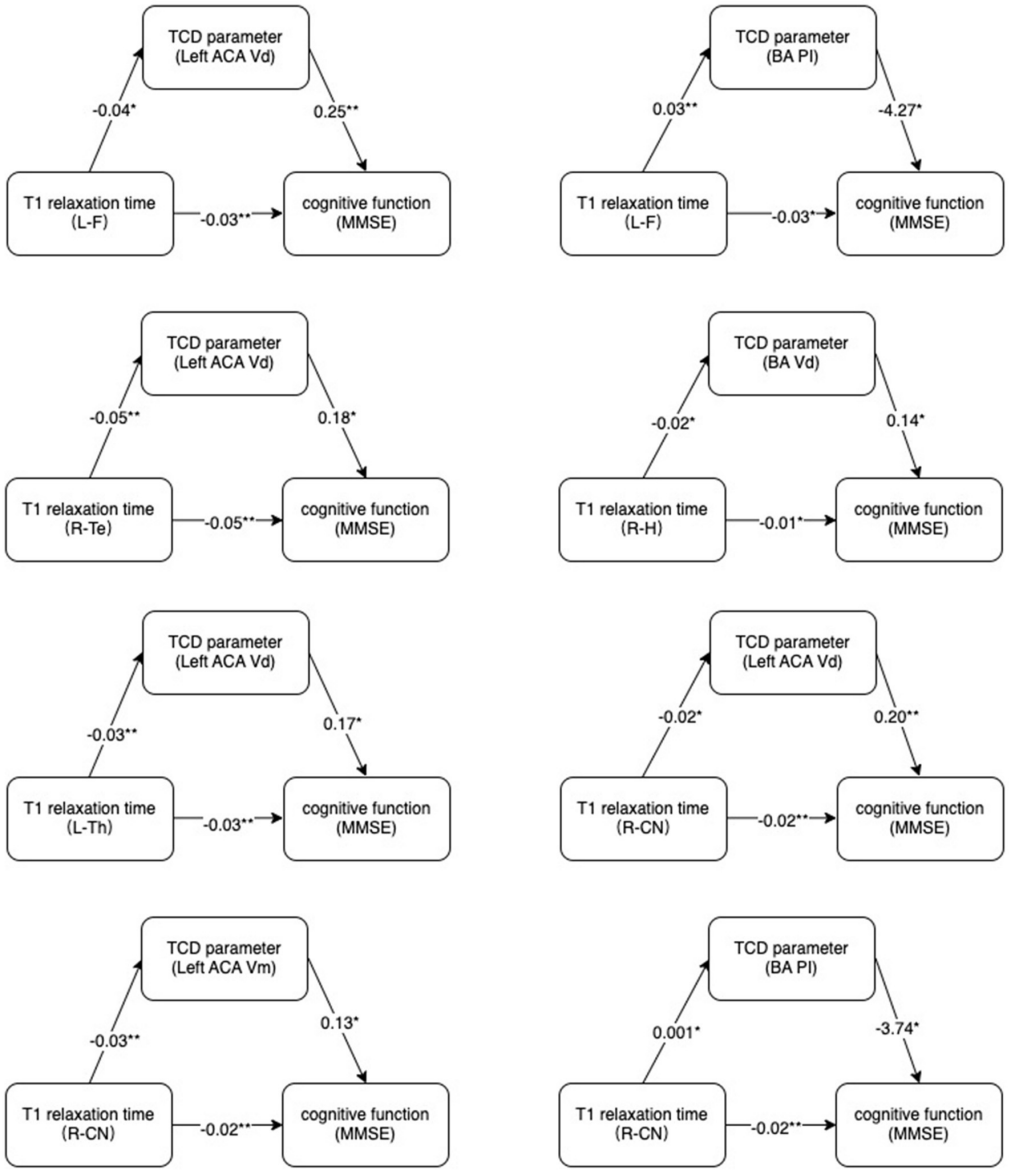
Mechanism diagram. Hypothesized direct and indirect pathways linking brain microstructure (T1 relaxation times) to cognitive impairment (MMSE) through a mediator (TCD parameters).MMSE, Mini-Mental State Examination. *p < 0.05, **p < 0.01.

The statistically significant T1 relaxation times were used as independent variables. MMSE scores were used as dependent variables. TCD parameters that correlated with both T1 relaxation times and MMSE were used as mediators. The results showed a potential correlation between T1 relaxation times and MMSE through cerebral TCD parameters (Figure 4).

## Discussion

Cognitive impairment commonly occurs after acute cerebral infarction. This cognitive impairment is influenced by a variety of factors. In order to avoid these interferences, the CI, N-CI, and Normal groups did not significantly differ from one another in terms of age, gender, years of education, vascular risk factors, or especially white matter lesions (WML). The impact of potential confounding factors on the evaluation of cognitive performance was substantially mitigated or abolished. Subcortical ischemic vascular disease is particularly prevalent in the general population, which includes white matter lesions. T2 sequences and Flair (fluid-attenuated inversion recovery) sequences were used to quantify white matter lesions using the Fazekas scale (21). The mechanism underlying the associations of WML with cognitive impairment after stroke is unclear. Previous literature demonstrated that white matter lesions impaired executive function and slowed processing speed, which increased the risk of cognitive impairment after stroke (21, 22). Another potential explanation was that WML may disrupt neuronal networks relevant to cognitive reserve and rehabilitation thereby affecting stroke prognosis (23).

Previous studies have clearly demonstrated that the site of cerebral infarction was strongly associated with cognitive impairment after stroke (24, 25). In our experiment, there were no significant differences in stroke location, size and severity between the CI group and the N-CI group, which largely eliminated the influence of cerebral infarction location on T1 relaxation times.

Structural imaging-based assessment of cognitive function alone has some limitations (26). We combined the MP2RAGE sequence, TCD technique and neuropsychological assessment to evaluate cognitive function, which was much more supportive in our diagnosis of the disease. Concerning TCD, a plethora of studies have investigated the connections between changes in cerebral hemodynamics and changes in cognitive function (20, 27, 28). One of the investigations mentioned above indicated that individuals with vascular cognitive impairment but no dementia reached the conclusion that most cerebral blood flow velocity was reduced while PI rose (20). According to our research findings, parts of the TCD parameters were considerably distinct among the three groups. There were statistically significant differences between the CI group and the normal group, which was mainly in line with the previous study's findings. But the N-CI and the Normal groups were compared, and no statistically significant difference was found between them. Previous studies have demonstrated that cerebral hemodynamics impairment is associated with the severity of stroke (29, 30), and not all stroke patients presented with significant cerebral hemodynamic impairment. Our results only showed significant cerebral hemodynamic impairment in patients with cognitive impairment after acute cerebral infarction. We think it may be caused by relatively higher demand for cerebral blood flow oxygen consumption in those patients. As a result, there were statistically significant differences between the CI group and the normal group.

T1 relaxation times primarily provide information about the myelin composition of the tissue and sensitively reflect injury to diffuse white and gray matter in the cranial brain (31). Earlier research conducted on patients suffering from neurological disorders found that their T1 relaxation times were significantly longer. T1 relaxation times tend to lengthen when pathological processes like myelin loss, axonal loss, and gliosis are present (32–34). T1 relaxation time has been utilized in several studies to evaluate changes in brain structure within neurological disorders, such as ischemic stroke or cognitive impairment (11, 35, 36). According to our research findings, the three different groups all had noticeably distinct T1 relaxation times for the aforementioned 8 brain regions. Therefore, this gave rise to the hypothesis that T1 relaxation times may be employed as a potential biomarker for identifying cognitive impairment after acute cerebral infarction.

It was generally understood that a stroke had an effect on the entirety of the brain as well as the features of its network rather than being a focal disease with restricted damage (37). In individuals who had cognitive impairment following an acute cerebral infarction, we found that varying T1 relaxation times were a phenomenon that occurred throughout the entire brain and were not confined to a particular brain region. The findings of our study confirmed this. Executive function, attention, psychomotor speed, and visual scanning are considered frontal lobe-related functions (38). In the literature, infarcts in the cortical region are more likely to lead to disruption of frontal-subcortical circuits, thereby disrupting local structures and functions within the networks that control cognitive functions. The temporal lobe is the primary region of the brain that is accountable for forming and maintaining long-term memories. The hippocampus plays a vital role in the process of learning. A recent study indicated that the precuneus is connected with numerous high levels of cognitive activities, including the processing of self-related information, situational memory, and visuospatial (39). The thalamus plays a vital role in various cognitive processes, including attention, executive ability, and working memory. The head of the caudate nucleus is an essential component of the cognitive circuit because it links the frontal lobe, the thalamus, the limbic system, and other structures; it also receives signals from frontal and temporoparietal regions bilaterally and sends out efferent fibers to the different areas of the basal ganglia (40). We considered that acute cerebral infarction caused the aforementioned microstructural disruption in 8 brain regions intimately connected to cognitive function. This microstructural disruption led to severe pathological myelin loss and axonal injury. There was a link between T1 relaxation times and cognitive performance, and this association could indicate, to some extent, the severity of cognitive impairment after acute cerebral infarction.

Our findings validated that the potential relationship between brain microstructural alterations and cognitive impairment could be explained by cerebral hemodynamics. Some studies showed that white matter microstructural change altered cerebral hemodynamic (41, 42). A possible mechanism was that pathological changes in the whole brain microstructure occurred after acute cerebral infarction. When the structural integrity of the white matter was disrupted, the compliance of the cerebral vessels was reduced, further causing cerebral hemodynamic disturbances. And hypoperfusion may lead to ischemia in various brain regions, resulting in cognitive impairment (43, 44). In addition, previous research has revealed that the blood-brain barrier is disrupted during the acute phase of cerebral infarction (45–47), followed by the deposition of substantial amounts of reactive oxygen species and circulating proteins in the brain (48, 49). These changes were not only limited to the brain region innervated by the damaged vessels, but also circulated throughout the brain with cerebral blood flow. Cerebral vasoconstriction, cerebral arteriosclerosis, and increased cerebral microvascular resistance were all produced by reactive oxygen species and circulating proteins, resulting in cerebral blood flow disorders (49). Endothelin and prostacyclin were the inflammatory cytokines generated during microangiopathy (50). These inflammatory cytokines interfered with the autoregulation of whole brain blood flow (51). All of the aforementioned pathophysiological alterations resulted in cerebral hypoperfusion. Cerebral hypoperfusion produced pathological myelin loss and axonal injury, altered nerve cell metabolism, promoted neurodegeneration, caused white matter bundle ischemia, disrupted subcortical circuits, and eventually resulted in abnormalities in brain tissue microstructure (20, 52, 53).

As well known, it has been confirmed that the right anterior temporal lobe has an important role in magnitude knowledge (54). In addition, there have been research confirming that the right temporal lobe was closely associated with visual memory impairment and verbal memory (55). In our study, ROC analysis demonstrated that AUC from T1 relaxation time on the right temporal lobe was biggest among all data, therefore it could be validated as a good individual discriminator of cognitive dysfunction after acute cerebral infarction in the future.

## Limitations

There were several limitations in this study as well. In the current study, patients with severe dementia who lacked informed capacity were excluded. Therefore, changes in T1 relaxation time did not represent a range of values for different levels of cognitive impairment. In the future, we will try to enroll these patients for further analysis in the study. In addition, we will try to carry out the research on the relation of scores on different aspects of cognition with T1 relaxation time. The content of various substances in brain tissue changes with time. Therefore, we inferred that T1 relaxation times would change consequently. In this experiment, we controlled for no statistical difference in the time interval between the stroke onset and imaging time among the three groups. In the future, we will conduct further cross-sectional analyses to compare the changes in T1 relaxation times of all ROIs at different examination times. Based on the definition of stroke, we will consider enrolling patients whose symptoms or signs lasted ≤ 24 h but with imaging revealing an ischemic lesion on the relevant side and location. The sample size of this study was modest, and it is expected that the sensitivity of T1 relaxation times in the diagnosis of cognitive impairment after acute cerebral infarction would be raised further.

## Conclusion

In conclusion, microstructural alterations in the whole brain occurred after acute cerebral infarction and could be identified by MP2RAGE sequences. Such microstructural alterations may contribute to cognitive impairment through changes in cerebral hemodynamics. T1 relaxation times on the right temporal lobe and the right frontal lobe are expected to be a biomarker of cognitive impairment after acute cerebral infarction.

## Data availability statement

The original contributions presented in the study are included in the article/supplementary material, further inquiries can be directed to the corresponding authors.

## Ethics statement

The studies involving human participants were reviewed and approved by the Ethics Committee of Second Affiliated Hospital of Dalian Medical University. The patients/participants provided their written informed consent to participate in this study. Written informed consent was obtained from the individual(s) for the publication of any potentially identifiable images or data included in this article.

## Author contributions

YG and HC conceptualized this study and designed this project. HY and YL performed the major procedures and wrote the manuscript. CW revised the manuscript and approved the final manuscript. HY, QZ, YG, YF, and HR contributed to the data collection. HY, YG, and HW assisted in the analysis of the data. All authors contributed to the article and approved the submitted version.

## Funding

This study was supported by the Natural Science Foundation of Liaoning Province (2022-MS-317), United Fund of the Second Hospital of Dalian Medical University and Dalian Institute of Chemical Physics, Chinese Academy of Sciences (UF-ZD-202012), and the 1+X program for Clinical Competency enhancement–Improvement of Clinical Technology Project, The Second Hospital of Dalian Medical University (2022LCJSGC04).

## Conflict of interest

The authors declare that the research was conducted in the absence of any commercial or financial relationships that could be construed as a potential conflict of interest.

## Publisher's note

All claims expressed in this article are solely those of the authors and do not necessarily represent those of their affiliated organizations, or those of the publisher, the editors and the reviewers. Any product that may be evaluated in this article, or claim that may be made by its manufacturer, is not guaranteed or endorsed by the publisher.
